# 

**Published:** 1889-09

**Authors:** 


### THE INTERNATIONAL MEDICAL CONGRESS.

We, the undersigned, do hereby give notice, that according to the resolution passed at the Washington meeting, Sept. 9th 1887, the

**10th International Medical Congress**

will be held in **Berlin.** —

The Congress will be opened on the 4th and closed on the 9th day of August 1890.

Detailed information as to the order of proceedings will be issued after the meeting of the delegates of the German Medical Faculties and Medical Societies at Heidelberg on the 17th of September in the current year.

Meanwhile, we should feel sincerely obliged, if you would kindly make this communication known among your medical circles and add in the same time our cordial invitation to the Congress.

von Bergmann. Virchow. Waldeyer.

### PUBLICATIONS RECEIVED.

From THE GAZETTE PRINTING CO., Montreal:
*Mc Gill University Annual Calendar. Faculty of Medicine.* 57th Session. 1889.
From 95 William Street, New York:
*The International Journal of Surgery.* June, July, 1889.
From P. BLAKISTON, SON & CO., Philadelphia:
*The Polyclinic.* June, 1889.
From F. A. DAVIS, Philadelphia:
*Lessons in Gynecology.* By WILLIAM GOODELL, A.M., M.D. Third Edition. 1887.
*American Resorts and Climates.* By BUSHROD W. JAMES, A.M., M.D. 1889.
From 3860 Pine Street, St. Louis:
*The Alienist and Neurologist.* July, 1889.
From FERET, Bordeaux:
*Annales de la Polyclinique de Bordeaux.* No. 2. Juillet, 1889.
From OCTAVE DOIN, Paris:
*Contribution a l'Étude des Corps étrangers des Voies aériennes.* Par le Dr. E. J. MOURE. 1889.
From 5 Avenue de l'Opéra, Paris:
*Annales d'Orthopédie et de Chirurgie practiques.* Nos. 12—17. 1889.
From EUGEN GROSSER, Berlin:
*Therapeutische Notizen der Deutschen Medizinal-Zeitung.* Herausgegeber Dr. JULIUS GROSSER. 1880—1889. Heft III. (Schlufsheft).
From JOHN WRIGHT & CO., Bristol:
*The Clinical Use of Prisms.* By ERNEST E. MADDOX, M.B. 1889.
From YOUNG J. PENTLAND, Edinburgh:
*Studies in Clinical Medicine.* By BYROM BRAMWELL, M.D. Vol I., Nos. 4—7. 1889.
From BAILLIÈRE, TINDALL & COX, London:
*Diseases of the Skin.* By HENRY S. PURDON, M.D. 1889.
From J. & A. CHURCHILL, London:
*On Bronchial Asthma.* By J. B. BERKART, M.D. Second Edition. 1889.
*Guide to B.P.* By PROSSER JAMES, M.D. Third Edition. 1889.
From H. K. LEWIS, London:
*The British Journal of Dermatology.* July—September, 1889.
*Gout.* By ROBSON ROOSE, M.D. Sixth Edition. 1889.
*Gynæcological Electro-Therapeutics.* By HORATIO R. BIGELOW, M.D. 1889.
*What to do in Cases of Poisoning.* By WILLIAM MURRELL, M.D. Sixth Edition. 1889.
*Bedside Urine-Testing.* By GEORGE OLIVER, M.D. Fourth Edition. 1889.
*Hygiene and Public Health.* By LOUIS C. PARKES, M.D. 1889.
From SAMPSON LOW, MARSTON, SEARLE & RIVINGTON, LIMITED, London:
*The Nursing Record.* June 27th, 1889.
From Messrs. RIVINGTON, London:
*Animal Biology.* By C. LLOYD MORGAN. 1889.
From JAMES WOOLLEY, SONS & CO., Manchester:
*Specialities and Recently Introduced Remedies.* Sixth Edition. 1889.

### *RECENT MEDICAL WORKS*

### PUBLISHED BY JOHN WRIGHT AND CO., BRISTOL.

---

*Demy 8vo, 546 pages, Cloth* 10/6, *post free. New Handy Edition of* PYE'S HANDICRAFT, *with* 233 *Illustrations. Revised throughout by the Author, and written up to date. Contains much new matter and many new illustrations.*

SURGICAL HANDICRAFT: A MANUAL OF SURGICAL MANIPULATIONS, MINOR SURGERY, ETC. For the use of General Practitioners, House Surgeons, Students and Surgical Dressers. By WALTER PYE, F.R.C.S., Surgeon to St. Mary's Hospital and the Victoria Hospital for Sick Children; late Examiner in Surgery at the University of Glasgow. With Special Chapters on AURAL SURGERY, TEETH EXTRACTION, ANÆSTHETICS, &c., by Messrs. FIELD, HOWARD HAYWARD, and MILLS.

EXTRACTS FROM PRESS NOTICES OF SECOND EDITION.

"Mr. Pye has reduced the size of his volume considerably, and has made some alterations in the text by excision and addition; he has thus added to its value, and also put it within the reach of a much wider circle of readers."—*The Lancet.*

"We had occasion to notice favourably the first edition of this manual of surgical manipulations, minor surgery, and other matters connected with the work of house-surgeons and surgical dressers. The work is a very practical and useful one, and the author is to be congratulated upon its success. The student, too, will not be sorry to find the present edition less costly, as well as more full of information, and also more portable. The book is certainly worthy of its author's reputation, and can be safely recommended."—*British Medical Journal.*

"Mr. Pye has written a volume full of interesting information, which is not confined to a mere haphazard chronicling of instruments ancient and modern. Throughout the work the author rigidly weighs the *pros* and *cons* of most that he alludes to, and by this means makes it eminently more readable and less formal. He very wisely dwells upon the importance of the faculty of improvisation, which is generally a fair indication of the practical man, in contradistinction to him whose strength lies in a good memory. To minutely review such a work as Mr. Pye's is akin to impossible by reason of its wide scope and variety of detail, but the student, house-surgeon, and junior practitioner will find by its perusal quite a store of useful facts and suggestions. The illustrations are numerous, excellent, and well chosen.—*Provincial Medical Journal.*

"A new edition of this practical work, in so short a time after the first, is strong evidence of its appreciation by the public, and is a source of congratulation to the author. It was reviewed in the *London Medical Record* (November, 1884,) and certain suggestions made in that review have been acted upon with, we think, manifest advantage to the work. The author has materially added to the usefulness of the book by slightly reducing its bulk, and yet, by using rather more condensed type, has been enabled to put more material into a smaller compass. The time which has elapsed since the first edition is too short to have been attended by any startling novelties in surgery, but Mr. Pye has made judicious use of recent additions to surgical literature, and the *Manual of Surgical Handicraft* is likely to prove an useful and popular addition to the student's library. The present edition is an advance upon the original one, and that is speaking very highly in its favour."—*London Medical Record.*

"Such a rapid sale of the first edition is in itself a strong testimony to the favour with which the work has been received by the profession. The book can be recommended as a really useful one, containing much practical information, described in a clear and interesting way. Recent authorities have been consulted, so as to bring the matter well up to date. The illustrations are excellent, and have all the vividness of good life portraits. The presence of special chapters on subjects not usually treated of in works of this kind is a distinct advantage. The present edition is bound in a smaller and more convenient form than its predecessor, and is cheaper. It is likely to meet with a rapid sale."—*Edinburgh Medical Journal.*

"The subjects cover the whole range of surgery. The book is an exceedingly good guide for those for whom it seems to be intended, and it furnishes not only them but the general medical practitioner with an excellent book of reference for any emergency which may arise. The matter is well chosen, the style is clear and pleasant, the illustrations are many of them new, and the methods of treatment described are fully up to date. It is different in its scope from any book on minor surgery with which we are acquainted, and it compares favourably with them all."—*New York Medical Journal.*

"This useful work is said to be especially designed for Hospital Internes. It could be studied with advantage by practitioners. The man who has his time fully occupied with the demands of an exacting practice is not apt to give much thought to surgical cases, save under very peculiar circumstances. It don't pay; the responsibility is too great—the remuneration too uncertain. Yet if it be some detail of dressing, some method of manipulating, or some of the new variations of daily procedure constantly coming out, about which he seeks information, the above work will be about the only one we know of that will satisfy his requirements." —*The Cleveland Medical Gazette.*

"We are pleased to greet this new work upon surgical dressings, the more especially as it is the most comprehensive work of the kind with which we are acquainted."—*American Western Medical Reporter.*

### *RECENT MEDICAL WORKS*

PUBLISHED BY JOHN WRIGHT AND CO., BRISTOL.

*Just Published.* 112 *pages*, 8*vo*, *with about Forty Original Diagrams. Price* 3/6, *post free.*

THE CLINICAL USE OF PRISMS, and the DECENTERING OF LENSES. A practical guide to the uses, numeration, and construction of Prisms and Prismatic Combinations, and the Centering of Spectacle Lenses. By ERNEST E. MADDOX, M.B., late Syme Surgical Fellow, Edinburgh.

*Just Published, Third Edition,* 6*th Thousand. Revised and Enlarged.* 2/-, *post free. Small* 8*vo, Pocket size, Cloth. Upwards of* 80 *Illustrations.*

ELEMENTARY BANDAGING AND SURGICAL DRESSING: with Directions concerning the IMMEDIATE TREATMENT OF CASES OF EMERGENCY. For Students, Dressers, Nurses, Ambulance Societies, &c., &c. Mostly condensed from "*Pye's Surgical Handicraft.*" By WALTER PYE, F.R.C.S., Surgeon to St. Mary's Hospital; late Examiner in Surgery at the University of Glasgow, &c. Has been adopted by the St. John Ambulance Association.

NOTICES TO THIRD EDITION.

"Within the short period of three years it has reached a third edition. . . . The directions throughout are concise, clear and judicious; the illustrations precise, definite, perspicuous. Every surgical student, dresser, or nurse should have a copy of this little book at hand, or in the pocket."—*The Provincial Medical Journal.*

"The popularity of Mr. Pye's *Manual* is proved by this third issue. The author writes well, the diagrams are clear, and the book itself is very small and portable, although the paper and type are good, which is rarely the case in publications constructed for the pocket."—*British Medical Journal, April* 13*th*, 1889.

"That it is a popular work, justly appreciated by those for whom it was written—dressers—is evident from the fact that the third edition is called for within three years of the original one."—*Hospital Gazette, March* 16*th*, 1889.

"One of the most useful little works for dressers and nurses. The information which it contains is exceedingly valuable in cases of emergency. The author truly says that it is 'a very little book,' but it is large in usefulness."—*Chemist and Druggist, April* 27*th*, 1889.

"The directions are clear and the illustrations are good."—*Lancet.*

"An immense amount of practically useful information, which we may safely say is not to be met with elsewhere in so handy and concise a form. . . . It is just the kind of subject-matter upon which a great many persons require information. . . . We can honestly recommend Mr. Pye's work to our readers as being carefully arranged and exceedingly well written throughout."—*Monthly Magazine of Pharmacy.*

"This useful little manual has already reached a third edition. It has evidently been appreciated by those dressers and nurses for whom it was first issued."—*Bookseller, April* 5*th*, 1889.

"A third edition of Mr. WALTER PYE's admirable little manual."—*Scotsman, March* 18*th*, 1889.

*Forty-Fifth Thousand.*

WRIGHT'S REGISTERED POCKET CHARTS for Bedside Case taking. Compiled by ROBERT SIMPSON, L.R.C.P., L.R.C.S. These Charts supply a long-felt want in the Profession by providing a method of recording important cases without loss of time and with little trouble. The advantages of the Pocket Charts over the Case Books in ordinary use are very great. PRICES, Post Free:—

| | |
|---|---|
| 50 CHARTS, folded for Pocket Case - - - | 2/6 |
| Ditto on Cards, Eyeleted Flat - - - | 6/- |
| 1 POCKET CASE, limp roan, to hold 50 Charts, complete with Indelible Ink Pencil - - - - | 2/6 |
| 1 GUARD BOOK, half bound, gold lettered, to hold 200 complete Charts - - - - - | 6/- |
| 50 CHARTS, 1 GUARD BOOK, and 1 POCKET CASE, complete | 10/6 |
| CHART HOLDERS, for hanging at bed heads, 1/- each or 9/- per doz. | |

*Special Quotations to Hospitals taking not less than* 250 *Charts, unfolded.*

"A very convenient arrangement. . . The price of the Charts, which are very complete, is most moderate."—*Brit. Med. Journal.*

1706.89

### *RECENT MEDICAL WORKS*

### Published by John Wright and Co., Bristol.

*Now Ready.* 8vo, 300 *pp., with Fifty High-class Original Illustrations. Price* 6/6, *post free.*

**LECTURES ON BRIGHT'S DISEASE.** By Robert Saundby, M.D. Edin., F.R.C.P. Lond., Physician to the General Hospital, Birmingham.

Press Notices.

"Dr. Saundby has long been known as a diligent and indefatigable worker in the field of renal pathology and therapeutics, so that these lectures will command for themselves a recognition beyond what any words of commendation can bestow. And, indeed, these lectures are full of good things, for, though not an "epoch-making" book, it is one of those works which throws a clear and steady light on doubtful and obscure questions, while arranging and classifying facts already accepted, but to which no recognised position had as yet been assigned. In other words, Dr. Saundby in these lectures has focussed for us the point to which our knowledge of the disease in question has reached, and placed that knowledge concisely and lucidly before us. . . . We say that these lectures should be attentively read by all who are desirous of gaining a clear insight into what is meant by Bright's Disease, and who may wish to follow intelligently the various changes and complications likely to occur in any given case under their care."—*Lancet.*

"We perused with enjoyment and profit this valuable work. . . . It presents in a small compact form the results of much careful work and hard thinking on a subject with which Dr. Saundby has peculiarly associated his name. We ought to add that a number of beautiful drawings illustrate the text."—*Practitioner.*

"It is a book which no practitioner should be without, as though it is so concise, yet there is no subject connected with albuminuria on which the most recent information is not to be had, together with a very full bibliography of all recent works on each branch of the subject, which those desirous of fuller information may consult. This bibliography has not been constructed from a library catalogue, but every work has evidently been well read and studied by the author, and is so referred to in the text that the student will have no difficulty in selecting the treatises that will be useful to him in his inquiries. . . . There is no ambiguity in regard to Saundby's opinions, which are enunciated with as much clearness and distinctness as brevity. . . . It would be difficult to point out another work in which the history, classification, and etiology of albuminuria is comprised in fifteen pages; and yet no one, we think, could rise from the perusal of these fifteen pages without having acquired clearer and more distinct views of the different pathological theories that have been propounded to account for the various forms of kidney disease from the days of Bright down to the present time. And yet these theories are so concisely described that no one, not in his dotage, is ever likely again to forget them. . . . We have great pleasure in recommending this book as a most handy and useful one for the consulting room, one which may be picked up and referred to on any subject connected with the diagnosis and treatment of albuminuria, with the certainty of obtaining the most recent information regarding it. It is beautifully printed, and the illustrations are distinct and accurate. It is altogether a work of which both author and publisher may be proud as a work of art."—*Edinburgh Medical Journal.*

"We have no hesitation in pronouncing the volume a most excellent one, and in every way deserving the confidence of the profession."—*Glasgow Medical Journal.*

"It is well known to the profession that Dr. Saundby has long been an earnest worker in the elucidation of questions connected with Bright's disease. We may say at once that we have read the book with great pleasure and profit, and we very warmly recommend its study by the profession. The size of the work is so modest (290 pages) that on that ground alone it invites perusal, and the volume recommends itself from the fact that it will easily find a nook on the library shelves. As might have been expected from Dr. Saundby, the matter, taken both from personal observation and wide general reading, is accurate, well digested, and concisely and perspicuously arranged. For those who wish to follow up the various points discussed there is a bibliography given at the end of each chapter, and the text is illustrated by many excellent and useful figures. . . . Considering, therefore, the prevalence of the diseases discussed, their importance, and the hazy notions held by many regarding them, we believe that this little work will supply a great want, and will meet with the large measure of success which it deserves. We may add that the book is most excellently and carefully printed."—*Illustrated Medical News, April 6th,* 1889.

"The author has long been known as an authority on everything pertaining to renal disease; and his views on renal pathology, and his methods of testing for albumen, are now accepted by those most competent to judge of such matters. . . . . A marked and useful feature of this book is the copious bibliography which follows each chapter, and which is well brought up to date. Dr. Saundby manages, however, in each chapter to give all the pith of the most important papers, and thus saves the reader the trouble of referring to the papers themselves. Being, as is well known, well posted in foreign medical literature, especially German, he is able to furnish his readers with information from the original sources, not at second-hand, as is but too often the case with text-book writers who sometimes are either ignorant of German or too lazy to take the trouble of verifying quotations. . . . . This book meets a long-felt want, and will, we believe, have as large a circulation as it deserves. The type and paper are very good, and, when necessary, original illustrations are introduced into the text." —*Birmingham Medical Review, May,* 1889.

"A work which gives, within a reasonable compass, all that is best in modern literature, supported at the same time by individual practical experience, so that it is a work which the conscientious general practitioner ought to have by him. We must congratulate the publishers on the excellent way the book has been produced."—***Provincial Medical Journal.***

P.T.O.

**BRIGHT'S DISEASE**—*continued.*

"The task of writing a new treatise on a subject which, in spite of the numerous researches made since Bright published his reports in 1827, has still many obscure points in its pathology and pathogenesis, is by no means an easy one. Dr. Saundby, however, has accomplished this task very ably, and has succeeded in putting together, in less than 300 pages, an admirable account of Bright's Disease in its various forms. It is so thoroughly practical that it recommends itself to the busy practitioner, and yet so scientific that even the specialist may consult its pages with great profit. Dr. Saundby's lectures are not merely compilations; the author has himself materially assisted in the unravelling of some of the many obscure points in the pathology of Bright's Disease, and has a large clinical material to draw upon for the illustration of his lectures. . . . Though the author's classification may be objected to, the description of the symptoms in the several cases which are given for illustration leaves nothing to be desired, and the same may be said of the chapter on treatment with which the work concludes. Dr. Saundby has succeeded in giving, in a clear and concise form, an accurate account of our present knowledge on albuminuria and Bright's Disease. The work is written in an easy and readable style, and the illustrations are numerous and well executed."—*British Medical Journal.*

"No such succinct and comprehensive *résumé* of the subject has ever appeared from any pen."—*Monthly Magazine of Pharmacy.*

"An original and most valuable contribution to the literature of kidney disease."—*Dublin Medical Journal.*

"It is a genuine pleasure to say that a book is a good one. . . . Without hesitation we can say Dr. Saundby's book is both solid and good. . . . Worth diligent study from cover to cover: and, except upon the one point of classification, there is nothing better extant in our language."—*Bristol Medical Journal.*

---

*Just Published.* 400 *pages,* 8*vo, with over* 60 *Illustrations. Price* 7/6, *post free.*

LECTURES ON MASSAGE AND ELECTRICITY IN THE CURATIVE TREATMENT OF DISEASE. By THOMAS STRETCH DOWSE, M.D., F.R.C.P. Edin. Formerly Phys. Supt. Central London Sick Asylum, Associate Member Neurological Society, New York.

PRESS NOTICES.

"Dr. Dowse's work is exceedingly opportune. Ably constructed, modestly written, and admirably expressed, from the first page to the last, this book is one which should be not only referred to, but attentively studied by all medical practitioners and students who can read English."—*The Monthly Magazine of Pharmacy.*

"Contains a vast number of important and practical reflections on the subject of massage and electricity. . . . The rapidity with which massage treatment has come up in the last few years endows a volume like this with extra importance."—*Science Gossip.*

"Massage has made rapid strides in medical favour of late years. The present excellent volume will do much to give it its rightful place in the medical armamentarium, and free it from the ignoble clutches of charlatans."—*Glasgow Herald.*

---

*Seventh Year.* 8*vo, Cloth Gilt, about* 700 *pages. Price* 6/6, *post free*

THE MEDICAL ANNUAL, 1889. Uniform in arrangement with former issues, containing ENTIRELY NEW MATTER, including important Original Contributions, designed to show the latest views and Treatment of every form of Disease, with a Synopsis of Therapeutic Agents, and their Clinical Indications.

"This 'Annual' is so full of live matter, and the dead limbs of last season have been so thoroughly pruned away, that it comes to the reader like a breath of fresh air medicated. . . . We feel sure the readers of the 'Annual' will coincide with us in thinking that, whether heretical or orthodox, the 'Annual' is a live, useful book, and deserves a place in every library."—*Philadelphia Therapeutic Gazette.*

"Can be unreservedly recommended to Practitioners."—*Lancet.*

"One of the most remarkable examples of publishing enterprise, and of its kind without an equal."—*Med. Press & Circ.*

"The edition for 1887 proved so popular that a reprint of it has lately been called for, and there can be no doubt that the work for the present year will also be widely referred to by medical men and pharmacists, not only in this country but abroad. The scope and the intention of the 'Annual' are alike admirable. . . . There are many admirably written articles to be found in it, calculated to be of the greatest value for reference to medical men and pharmacists. . . . Generally speaking, the articles are both concise and comprehensive, and we shall gladly abstract one or two of these here as samples of the rest."—*Provincial Medical Journal.*

"Year by year this volume is growing in size and importance, and the long list of editors of the present edition, many of them men well known in the profession, is sufficient guarantee of the reliability and value of the volume. Every general practitioner should have it on his study table."—*Glasgow Medical Journal.*

"The annual is now in its seventh year of issue. . . . It is as great a help as ever to the busy practitioner."—*Edinburgh Medical Journal.*

THE INTERNATIONAL MEDICAL CONGRESS ... ... ... ... ... ... ... ... 214

NOTES ON PREPARATIONS FOR THE SICK ... ... ... ... ... ... ... ... 215

PERISCOPE ... ... ... ... ... ... ... ... ... ... ... ... ... ... ... ... 217

SCRAPS ... ... ... ... ... ... ... ... ... ... ... ... ... ... ... ... ... 223

*Cases for binding, price 7d. each, may be had of the Publisher,*
J. W. ARROWSMITH, *Quay Street, Bristol.*

